# Multivariate analysis of oestrogen receptor alpha, pS2, metallothionein and CD24 expression in invasive breast cancers

**DOI:** 10.1038/sj.bjc.6603254

**Published:** 2006-08-01

**Authors:** P Surowiak, V Materna, B Györffy, R Matkowski, A Wojnar, A Maciejczyk, P Paluchowski, P Dzięgiel, M Pudełko, J Kornafel, M Dietel, G Kristiansen, M Zabel, H Lage

**Affiliations:** 1 Institute of Pathology, Charité Campus Mitte, Schumannstr. 20/21, D-10117 Berlin, Germany; 2 Department of Histology and Embryology, University School of Medicine, ul. Chałubińskiego 6a, 50-356 Wrocław, Poland; 3 Lower Silesian Centre of Oncology, pl. Hirszfelda 12, 53-413 Wrocław, Poland; 4 Semmelweis University Budapest, Szentágothai János Knowledge Centre, Bókay u. 53/54, Budapest, H-1088 Hungary; 5 Pinneberg Hospital, Breast Centre, Fahltskamp 74, 25421 Pinneberg, Germany; 6 Department of Oncology, University School of Medicine, pl. Hirszfelda 12, 53-413 Wrocław, Poland; 7 Department of Histology and Embryology, University School of Medicine, ul. Święcickiego 6, 60-781 Poznań, Poland

**Keywords:** breast cancer, prognosis, oestrogen receptor alpha, pS2, metallothionein, CD24

## Abstract

Determination of oestrogen receptor alpha (ER) represents at present the most important predictive factor in breast cancers. Data of ours and of other authors suggest that promising predictive/prognostic factors may also include pS2, metallothionein (MT) and CD24. Present study aimed at determining prognostic and predictive value of immunohistochemical determination of ER, pS2, MT, and CD24 expression in sections originating from 104 patients with breast cancer. An univariate and multivariate analysis was performed. Both univariate and multivariate analyses demonstrated that cytoplasmic-membranous expression of CD24 (CD24c-m) represents a strong unfavourable prognostic factor in the entire group and in most of the subgroups of patients. In several subgroups of the patients also a prognostic value was demonstrated of elevated expression of pS2 and of membranous expression of CD24. Our studies demonstrated that all patients with good prognostic factors (higher ER and pS2 expressions, lower MT expression, CD24c-m negativity) survived total period of observation (103 months). The study documented that cytoplasmic-membranous expression of CD24 represented an extremely strong unfavourable prognostic factor in breast cancer. Examination of the entire panel of the studied proteins permitted to select a group of patients of an exceptionally good prognosis.

In the entire world, breast cancer represents the most frequent female malignant tumour. In 2002, 1 151 298 new cases of breast cancer were diagnosed and 410 712 deaths due to the disease were noted (Jemal *et al*, 2002). The principal therapeutic approach in breast cancer involves surgery. In advanced cases supplementary therapy is needed, involving pharmacotherapy and/or radiotherapy. Considering that breast cancer is a hormone-related tumour, cases of tumour with oestrogen receptor alpha (ER), or around 60% of breast cancer cases in postmenopausal women are treated with a drug of antioestrogenic activity, tamoxifen. Also other methods of hormonal treatment are in use, including aromatase inhibitors, analogues of GnRH, etc. In advanced cases of breast cancer, in cases of a more aggressive course or in cases insensitive to hormonal therapy chemotherapy is used (Goldhirsch *et al*, 1998; National Institutes of Health Consensus Development Panel, 2001; Mori *et al*, 2002). As the hormonal treatment, as compared to chemotherapy, is much better tolerated by the patients the capacity to predict clinical response to tamoxifen and clinical course of the tumour becomes a significant aim in the diagnosis.

In ER-positive cases, clinical response to tamoxifen treatment used to be obtained in only around 60% patients (Goldhirsch *et al*, 1998). Therefore, additional exponents of tamoxifen sensitivity/resistance and variables typical for breast cancer cases of a more aggressive course are searched for. Numerous studies have been focused on oestrogen-dependent proteins, that is, the proteins with ER-controlled expression. Coexpression of ER and of progesteron receptor was found to be typical for cases of a slower course and more sensitive to tamoxifen (Fitzgibbons *et al*, 2000). Another oestrogen-dependent protein involves pS2 (Nunez *et al*, 1987). However, data on prognostic value of the protein are equivocal and the problem requires further studies (Fitzgibbons *et al*, 2000; Surowiak *et al*, 2001). In our earlier studies, performed on a group of 60 patients with ductal breast cancer, postoperatively treated with tamoxifen, expression of metallothionein (MT), which is downregulated by ER was found to be an unfavourable prognostic factor (Surowiak *et al*, 2005b). In the same group of patients cytoplasmic/membraneous expression of CD24, which is also downregulated by ER, proved to be typical for cases with shorter overall survival time (Surowiak *et al*, 2006).

Prognostic and predictive value of novel unfavourable prognostic and predictive factors in breast cancer should be corroborated on a larger group of patients and should be compared to the significance of expression of the most important prognostic protein in the tumour, the oestrogen receptor. In order to corroborate significance of expression of the discussed proteins, also a multivariate analysis should be performed.

In this study, using univariate and multivariate analyses, prognostic and predictive values were estimated of immunohistochemical tests of ER, pS2, MT and CD24 expression in an unselected group of 104 patients with ductal breast cancer.

## MATERIALS AND METHODS

### Patients

Immunohistochemical analysis was performed retrospectively on tissue samples that were taken for routine diagnostic purposes. The cases were selected based on availability of tissue and were not stratified for known preoperative or pathological prognostic factors. The study was approved by an Institutional Review Board (IRB) and the patients gave their informed consent before their inclusion to the study. The total of 104 patients with primary invasive breast cancer, who were diagnosed in the years 1993–1994 in Lower Silesian Centre of Oncology in Wrocław, Poland, were qualified to the studies. All the patients were subjected to mastectomy and, then, were treated with radiotherapy and/or chemotherapy and/or hormonotherapy (Table 1). The patients were monitored by periodic medical check-ups, ultrasonographic and radiological examinations. During the follow-up period, 23 patients (22%) had a recurrent disease and 25 patients (24%) died of the disease. The mean progression-free survival time was 76 months (range 8–103 months) while the mean overall survival time was 81 months (range 8–103 months).

Fragments sampled from studied tumours were fixed in 10% buffered formalin and, then, embedded in paraffin. In every case, haematoxylin and eosin stained preparations were subjected to histopathological evaluation by two pathologists. The stage of tumours was assessed according to TNM classification system (Sobin and Wittekind, 2002). Tumour grade was estimated according to Bloom-Richardson in the modification of Elston and Ellis (Elston and Ellis, 1991) (Table 1).

### Immunohistochemistry

Formalin-fixed, paraffin-embedded tissue was freshly cut (4 *μ*m). The sections were mounted on Superfrost slides (Menzel Gläser, Germany), dewaxed with xylene and gradually hydrated. Activity of endogenous peroxidase was blocked by 5 min exposure to 3% H_2_O_2_. Detection of ER and CD24 expression was preceded by 15 min exposure of the sections in a microwave oven to boiling Antigen Retrieval Solution (DakoCytomation, Denmark) at 250 W. Then, immunohistochemical reactions were performed using the mouse monoclonal (clone 1D5) antibodies to ER (optimaly prediluted) (DakoCytomation, Denmark), polyclonal rabbit antibodies directed against pS2 (dilution 1 : 200) (Novocastra, UK), mouse monoclonal (clone E9) antibodies to MT (dilution 1 : 100) (DakoCytomation, Denmark) and the monoclonal (clone SN3) mouse antibodies detecting CD24 (dilution 1 : 100) (DakoCytomation, Denmark). The antibodies were diluted in the Antibody Diluent, Background Reducing (DakoCytomation, Denmark). The sections were incubated with an antibody for 1 h at room temperature. Subsequently, incubations were performed with biotinylated antibodies (15 min, room temperature) and with streptavidin-biotinylated peroxidase complex (15 min, room temperature) (LSAB2, HRP, DakoCytomation, Denmark). DAB (DakoCytomation, Denmark) was used as a chromogen (7 min, room temperature). All the sections were counterstained with Meyer's haematoxylin. In every case, controls were included in which specific antibody was substituted by the Primary Negative Control (DakoCytomation, Denmark).

### Evaluation of reaction intensity

Intensity of immunohistochemical reactions was evaluated independently by two pathologists. In equivocal cases, the preparation was re-evaluated in common. In cases of ER, pS2 and MT intensity of the immunocytochemical reactions was evaluated using the semiquantitative scale of the ImmunoReactive Score (IRS) (Remmele and Stegner, 1987), which took into account intensity of the colour reaction (0 – no reaction, 1 – weak reaction, 2 – moderate intensity, 3 – intense reaction) as well as proportion of positive cells (0 – no positive cells, 1 – <10% positive cells, 2 – 10–50% positive cells, 3 – 51–80% positive cells, 4 – >80% positive cells). The final score represented the product of points given for individual characters and ranged between 0 and 12. In evaluation of CD24 expression intensity the scale was employed, which took into account location of the reaction: the membranous (CD24m) or cytoplasmic-membranous one (CD24c-m). Cases with no CD24 expression or the expression in <10% cells were denoted by ‘0’ while cases with presence of CD24 in more than 10% of cancer cells were marked by ‘1’ (Surowiak *et al*, 2005a; Surowiak *et al*, 2006).

### Statistical analysis

Statistical analysis of the results took advantage of Statistica 98 PL software (Statsoft, Poland). The employed tests included *χ*^2^ test and the Spearman's rank correlation. Kaplan–Meier's statistics and log-rank tests were performed using SPSS software (release 10.0; SPSS Inc., Chicago, IL, USA) to estimate significance of differences in survival times. The length of survival was defined as the time between the primary surgical treatment and diagnosis of a recurrent tumour or death due to the neoplastic disease. We also performed analyses in the subgroups of the patients – treated with tamoxifen, treated without tamoxifen, treated with chemotherapy, treated without chemotherapy, treated with radiotherapy and treated without radiotherapy.

The multivariate analysis was performed using Cox regression test.

## RESULTS

### Immunostaining

**Figure 1:**
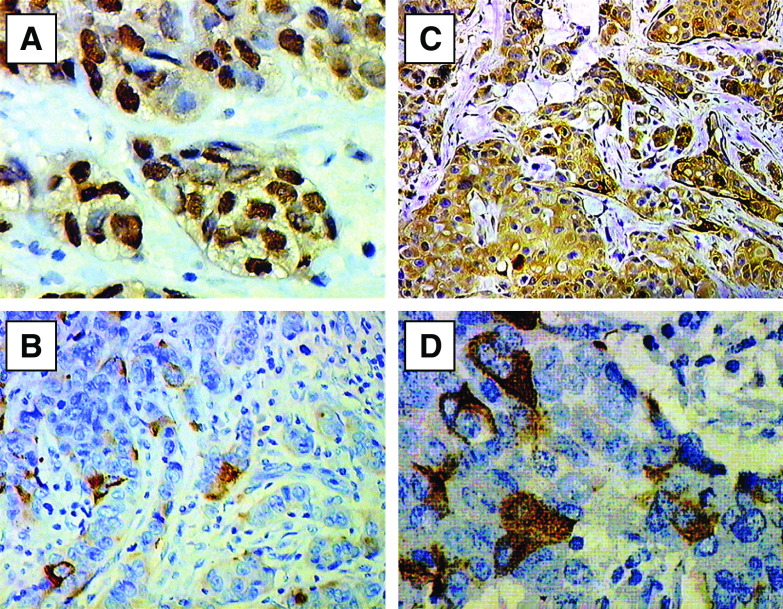
Immunohistochemical staining for: (**A**) oestrogen receptor alpha (haematoxylin, × 400), (**B**) pS2 (haematoxylin, × 200), (**C**) metallothionein (haematoxylin, × 200) and (**D**) cytoplasmic-membranous CD24 (haematoxylin, × 400) in the breast cancer specimens.

In the case of ER the obtained colour reaction was localized in cell nuclei (Figure 1A). Intensity of the reaction varied in individual cases. Mean immunoreactivity score amounted to 3.41±2.99 s.d.

In the case of pS2, we obtained a colour reaction localised in the cytoplasm of cancer cells (Figure 1B). The reaction varied in individual cases. The mean pS2 immunoreactivity score was 2.95±2.54 s.d.

In the case of MT, the immunohistochemical reactions yielded a colour reaction localized in the cytoplasm as well as in cell nuclei of cancer cells. The reaction varied in individual cases (Figure 1C). The mean MT immunoreactivity score was 6.06±2.97 s.d. The reaction was also found in the myoepithelial cells (Figure 1C).

The immunohistochemical reactions using antibodies directed to CD24 yielded in cancer cells colour reactions of a membranous localisation (CD24m) or a membranous-cytoplasmatic localisation (CD24c-m)(Figure 1D), of a variable intensity in individual cases. In 44 (42%) cases expression of CD24m was disclosed and in 44 (42%) cases the expression manifested the CD24c-m pattern.

### Studied protein expression and clinicopathological data

**Figure 2:**
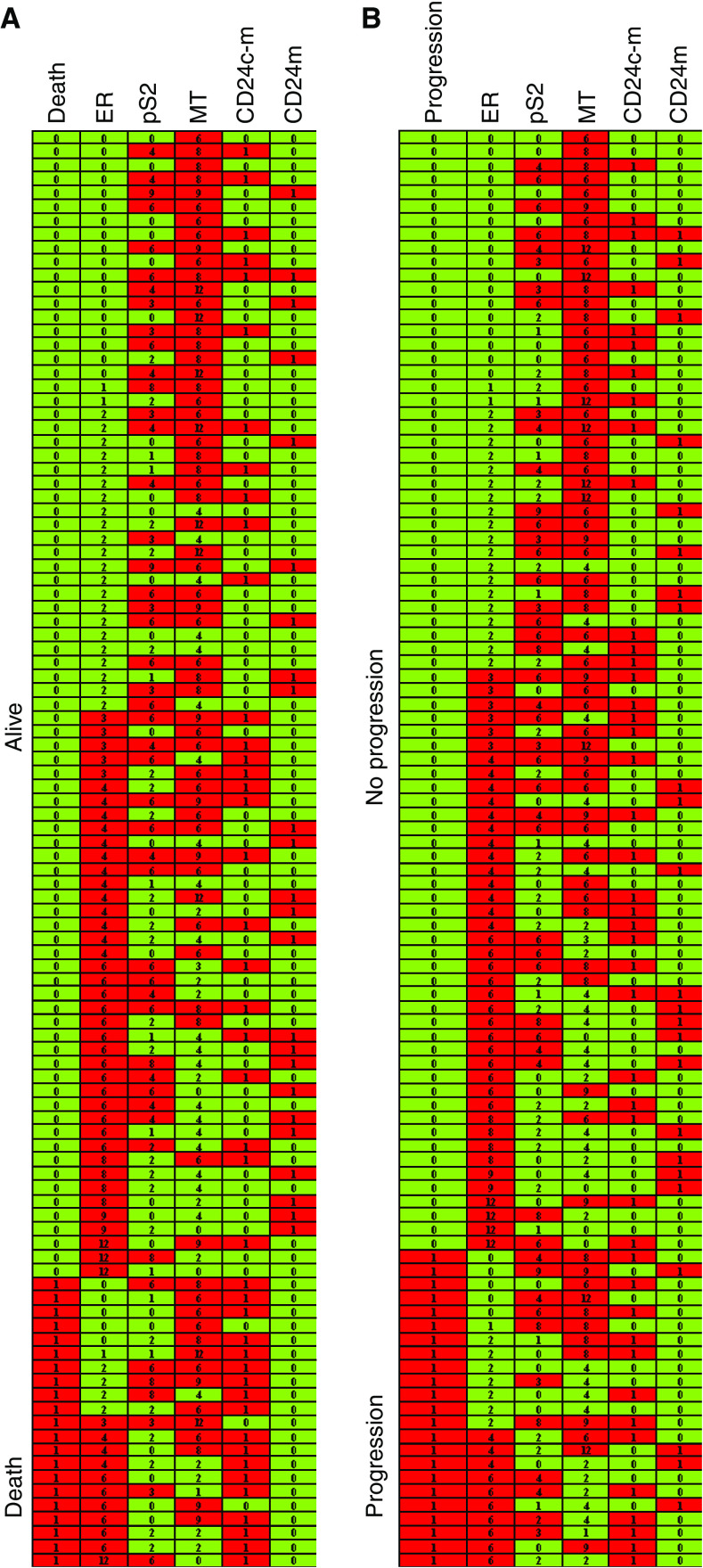
Expression of oestrogen receptor alpha (ER), pS2, metallothionein (MT), cytoplasmic-membranous CD24 (CD24c-m) and membranous CD24 (CD24 m) *vs* (**A**) survival of studied patients and (**B**) progression of the disease. The data are grouped according to expression of ER and: (**A**) survival and (**B**) progression.

Using the *χ*^2^ test relationships were examined between the ER, pS2, MT, CD24c-m and CD24m expression on one hand and the variables of the patients, including pT, pN, stage, grade, age (Spearman's rank correlation), menopause status, progression and deaths on the other. The tests proved that progression and deaths due to breast cancer were significantly more frequent in patients manifesting expression CD24c-m (Table 2) (Figure 2). Lower pS2 expression was found to be typical for premenopausal patients, cases of cancer of a higher grade and for patients with progression of the neoplastic disease (at the verge of statistical significance) (Table 2) (Figure 2).

### Univariate analysis in the entire studied group and in the subgroups

Univariate analysis demonstrated no relationship between menopausal status, grade, pT, PN and stage of the studied tumours on one hand and duration of survival of the patients (Table 1).

**Figure 3:**
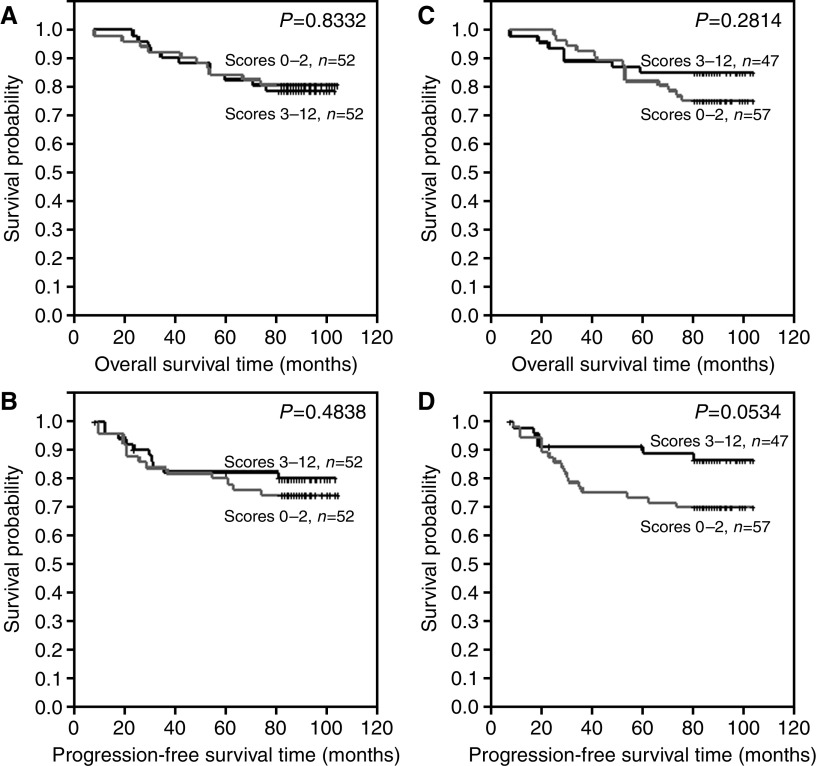
Kaplan–Meier curves for: (**A**) overall survival and oestrogen receptor alpha expression, (**B**) progression-free survival and oestrogen receptor alpha expression, (**C**) overall survival and pS2 expression, (**D**) progression-free survival and pS2 expression.

**Figure 4:**
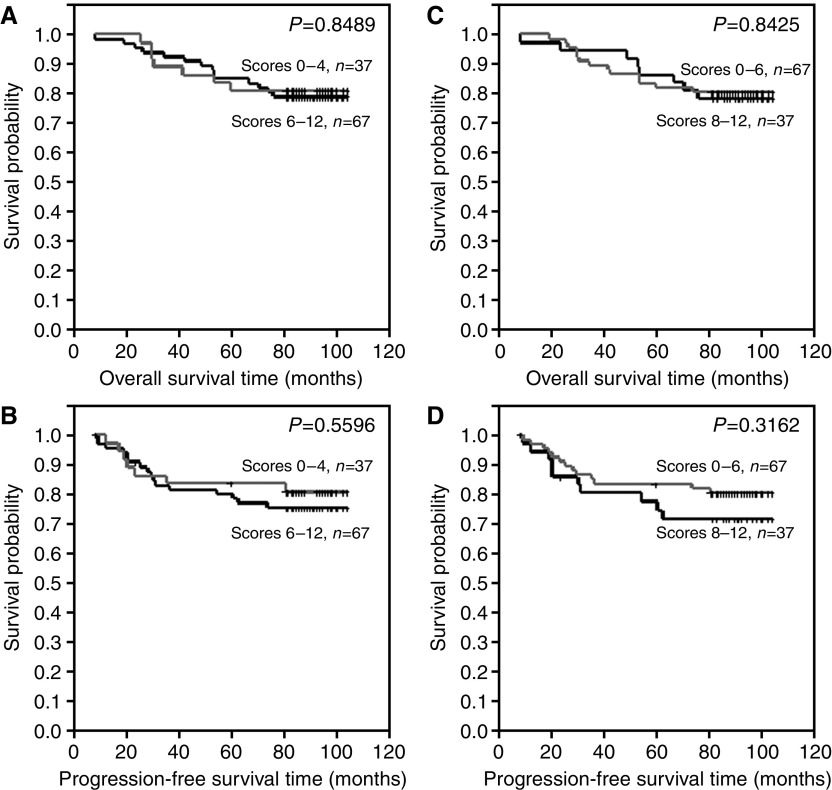
Kaplan–Meier curves for: (**A**, **C**) overall survival and metallothionein expression, (**B**, **D**) progression-free survival and metallothionein expression.

**Figure 5:**
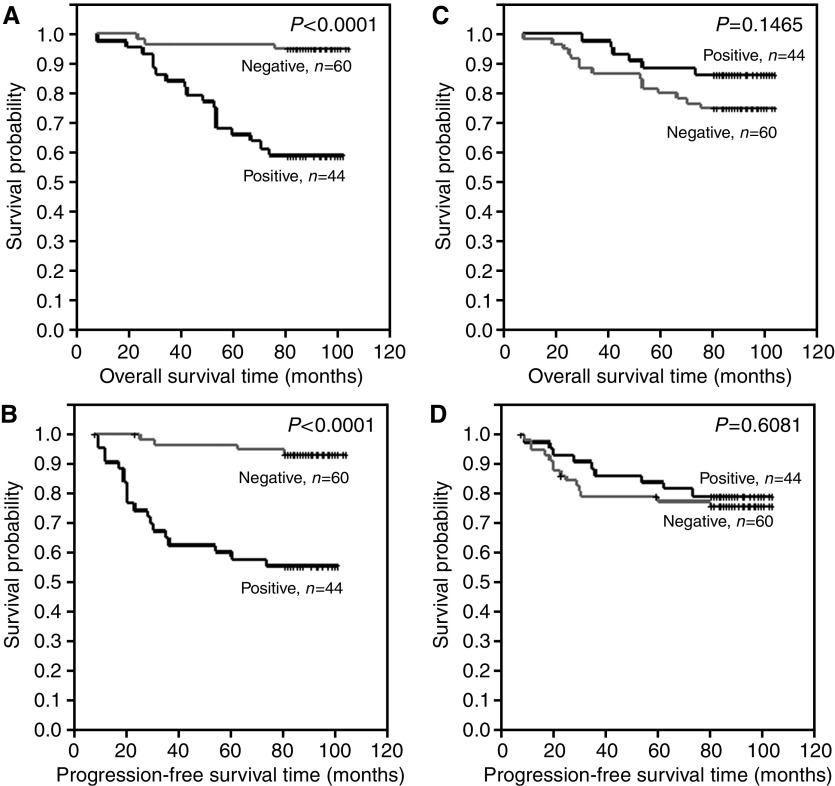
Kaplan–Meier curves for: (**A**) overall survival and cytoplasmic-membranous CD24 expression, (**B**) progression-free survival and cytoplasmic-membranous CD24 expression, (**C**) overall survival and membranous CD24 expression, (**D**) progression-free survival and membranous CD24 expression.

Differences in overall survival time and progression-free time were examined between the following groups: (A) patients with no or lower ER expression (IRS 0–2) and patients with higher ER expression (IRS 3–12) (Figure 3A and B), (B) patients with of no or lower pS2 expression (IRS 0–2) and patients with higher pS2 expression (IRS 3–12) (Figure 3C and D), (C) patients with no or lower MT expression (IRS 0–4) and patients with higher pS2 expression (IRS 6–12) (Figure 4A and B), (D) patients with no or lower MT expression (IRS 0–6) and patients with higher pS2 expression (IRS 8–12) (Figure 4C and D), (E) patients with no CD24c-m expression and patients with such expression (Figure 5A and B) and (F) patients with no CD24m expression and patients with such expression (Figure 5C and D). The tests demonstrated that CD24c-m positive cases exhibited a significantly shorter overall survival time and progression-free time (Figure 5A and B).

**Figure 6:**
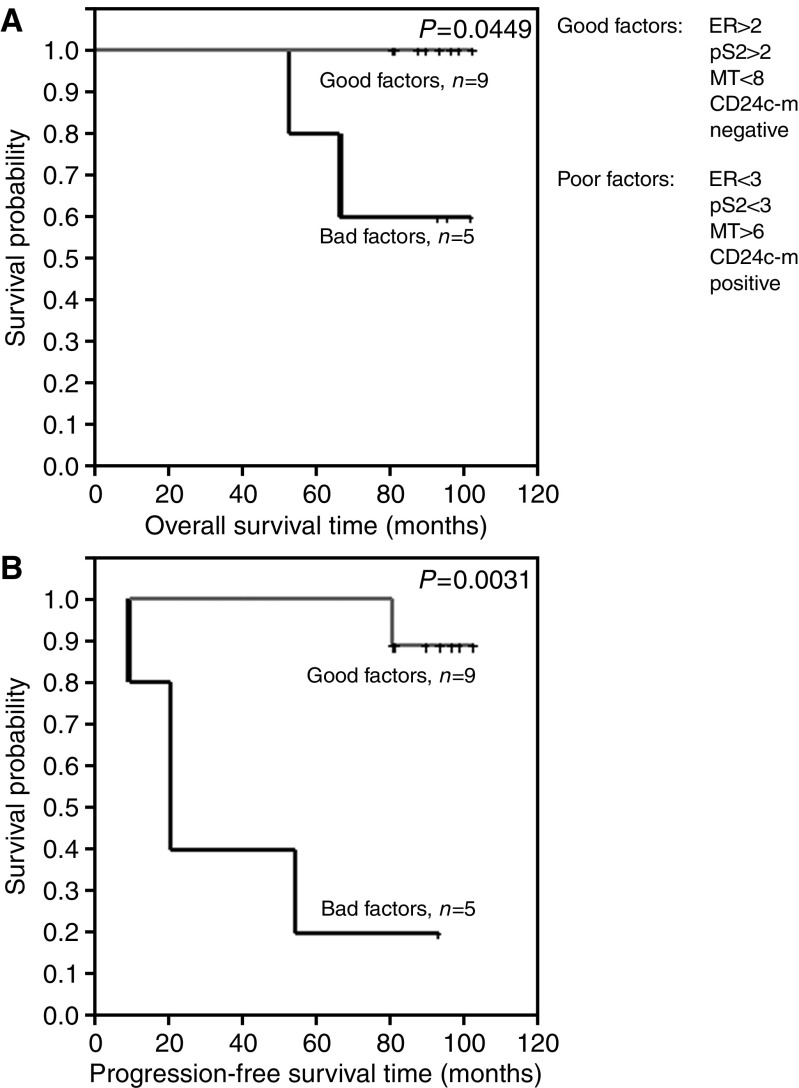
Kaplan–Meier curves for: (**A**) overall survival and expression of favourable or unfavourable prognostic indices, (**B**) progression-free survival and expression of favourable or unfavourable prognostic indices.

In turn, the differences in overall survival time and progression-free time were examined between patients with favourable prognostic factors (ER>2, pS2>2, MT<8, CD24c-m negative) and those with unfavourable prognostic factors (ER<3, *P*S2<3, MT>6, CD24c-m positive). Despite the very low numerical force of the compared groups (*n*=14) patients with favourable prognostic factors demonstrated a significantly longer overall survival time and progression-free survival (Figure 6). None of the patients with favourable prognostic factors died in the course of the observation.

We also performed analyses on the subgroups of the patients – treated with tamoxifen, treated without tamoxifen, treated with chemotherapy, treated without chemotherapy, treated with radiotherapy and treated without radiotherapy. In all cases of the subgroups CD24c-m positive patients were found to exhibit a significantly shorter overall survival time and progression-free survival (Table 3). Patients with lower expression of pS2 manifested a significantly shorter progression-free survival in the subgroup treated with tamoxifen and in the subgroup treated with radiotherapy (Table 3).

### Multivariate analysis

Using the Cox regression test relationships were examined between expression of studied proteins on one hand and overall survival time and progression-free survival on the other. The relations were examined both in the entire group of patients and in the subgroup of patients with various types of postsurgery therapy. The tests demonstrated that the CD24c-m positive cases manifested significantly shorter overall survival time and progression-free survival in the entire group as well as in the subgroups of patients treated with tamoxifen, treated with radiotherapy, treated without chemotherapy (Table 4). The patients with a lower pS2 expression manifested a significantly shorter progression-free survival in the subgroups of treated with tamoxifen and treated with radiotherapy (Table 4). CD24m positive patients exhibited a significantly longer overall survival time in the entire group as well as in the subgroups treated with tamoxifen and treated without chemotherapy (Table 4), while a significantly longer progression-free survival was detected in the subgroups treated with tamoxifen and treated with radiotherapy (Table 4).

## DISCUSSION

In the study, prognostic and predictive values have been examined of immunohistochemical studies on expression of four distinct proteins, linked either to sensitivity or to resistance of breast cancer cells to tamoxifen: oestrogen receptor alpha (ER), pS2 (Fitzgibbons *et al*, 2000), metallothionein (MT) (Surowiak *et al*, 2005b) and CD24 (Surowiak *et al*, 2006) in the group of 104 patients with invasive breast cancer. The relationship has also been examined between overall survival time and progression-free survival and the following clinical and pathological variables of studied patients or tumours: menopausal status, grade, pT, pN and stage.

Menopausal status, grade, pT, pN and stage belong to the most important prognostic indices in breast cancer (Goldhirsch *et al*, 1998; Fitzgibbons *et al*, 2000). Nevertheless, in this study no relationship could have been demonstrated between the variables on one hand and overall survival time and progression-free survival on the other. This has probably reflected the highly uniform character of the studied group: 96% of studied patients carried stage II of the tumour (38% with stage IIa and 58% with stage IIb).

Determination of ER expression belongs to the routine histopathological examination in cases of breast cancer. Oestrogen receptor represents at present the most significant predictive factors as related to subsequent results of tamoxifen treatment (Goldhirsch *et al*, 1998; Yoshinari *et al*, 1999; Fitzgibbons *et al*, 2000). In this study, such a prognostic significance of ER expression could not have been documented using either univariate analysis or multivariate analysis. Similarly, no significant effects of ER expression on survival rates could have been noted in subgroups of the patients treated with tamoxifen, treated without tamoxifen, treated with chemotherapy, treated without chemotherapy, treated with radiotherapy and treated without radiotherapy. The result might be regarded controversial but in the years of 1993–1994 ER expression was not yet routinely examined in the Lower Silesian Centre of Oncology (Wrocław, Poland) and, therefore, tamoxifen treatment of the patients was not applied in the targeted manner. In the group not treated with tamoxifen, mean intensity of ER expression in IRS scale was 3.41±2.26 s.d., and in the group treated with tamoxifen it amounted to 3.41±2.87 s.d. The group not treated with tamoxifen included 17 ER negative patients (IRS 0–2) and 17 ER positive patients (IRS 3–12). The group treated with tamoxifen included the same ratio of 35 ER negative and 35 ER positive patients. The phenomenon seemed to explain well the lack of relationship between ER expression and survival rates in the subgroup of tamoxifen-treated patients.

Literature data on prognostic/predictive value of pS2 protein are inconsistent (Fitzgibbons *et al*, 2000). In present study, we have demonstrated that elevated expression of pS2 is typical for postmenopausal patients with tumours of lower grades, in whom no progression of the neoplastic process has developed. Univariate analysis has permitted to show that elevated pS2 expression is typical of patients with extended progression-free survival in subgroups treated with tamoxifen and patients treated with radiotherapy. Similar data have been obtained using multivariate analysis. The results suggest that elevated expression of pS2 characterizes breast cancer cases of a more benign course. It should be stressed that no significant relationships of the type have been obtained for ER and, thus, pS2 seems to represent a strong favourable prognostic index.

In our previous study, elevated expression of MT was found to characterize cases resistant to tamoxifen treatment (Surowiak *et al*, 2005b). In present study, conducted on a higher number of unselected patients no relationship could have been demonstrated between MT expression and any clinical or pathological variables of the patients.

In our earlier studies the cytoplasmic/membranous expression of CD24 (CD24c-m) was found to represent an unfavourable prognostic index of breast cancer (Kristiansen *et al*, 2003c). In the subsequent studies performed on 60 tamoxifen-treated patients, we confirmed the unfavourable prognosis associated with CD24c-m expression (Surowiak *et al*, 2006). Presence of CD24 in cytoplasm of tumour cells has been explained by stimulated synthesis of the protein. Thus, in CD24c-m positive cases most probably presence of CD24 has been observed in its typical localisation, in the cell membrane, and the protein has been transported from endoplasmic reticulum. The observation has seemed to be confirmed by much higher intensity of the reaction in CD24c-m positive cases as compared to CD24m positive cases. In present study, we have corroborated the highly unfavourable prognostic value of CD24c-m. Using univariate analysis, the CD24c-m expression has been noted to correlate with shorter overall survival and progression-free survival in the entire group of the patients as well as in all the subgroups except of the patients not treated using radiotherapy. Multivariate analysis has allowed to confirm the unfavourable prognosis accompanying CD24c-m expression in the entire group as well as in subgroups of patients treated with tamoxifen, treated by radiotherapy and not treated with chemotherapy. The prognostically negative significance of CD24 expression has been linked to augmented invasive potential of cells manifesting expression of the protein. CD24 has been identified as the ligand of *P*-selectin, the adhesive receptor of endothelial cells and blood platelets. Most probably, CD24 facilitates intravasation of tumour cells (Kristiansen *et al*, 2002; Kristiansen *et al*, 2003a, 2003b, 2003c; Kristiansen *et al*, 2004). Recently, induction of CD24 expression in breast cancer cells was found to stimulate their proliferation and numerous invasive properties, like motility and aggressiveness (Baumann *et al*, 2005). Thus, the unfavourable significance of CD24 expression in breast cancer cells may be explained not only by augmented transfer through the vascular walls but also by the higher proliferative potential of the cells and their increased ability to invade intercellular matrix. Data of ours and of other authors suggest that CD24c-m represents one of the most significant prognostic indices in breast cancer.

In earlier studies, we were unable to link membranous expression of CD24 (CD24m) with survival of the patients (Surowiak *et al*, 2005a; Surowiak *et al*, 2006). Using multivariate analysis, in present study, we have demonstrated that CD24m positive cases manifested a significantly longer overall survival time in the entire group and in the subgroups treated with tamoxifen and treated without chemotherapy, and a significantly longer progression-free survival in the subgroups treated with tamoxifen and treated with radiotherapy. It should be noted that CD24c-m expression corresponds to higher intensity of CD24 expression and that the link between CD24c-m expression and survival rates of the studied patients was much stronger than that between CD24 m expression and the survival. The favourable prognostic value of CD24 m expression can be explained by the fact that the latter could have been noted in CD24c-m negative cases. The relationship indirectly reflects the fact that CD24 m positive cases are CD24c-m negative.

In the study, we have examined also differences in survival rates between the group of patients with favourable prognostic indices (ER>2, pS2>2, MT<8, CD24c-m negative) and the group showing unfavourable prognostic indices (ER<3, *P*S2<3, MT>6, CD24c-m positive). The result has been surprising: none of the patients with favourable prognostic indices died. Despite the low numerical force of the studied subgroup (the total of 14 patients) significant relationships could have been documented with the overall survival time and progression-free survival. Thus, examination of the panel of prognostic indices may significantly augment prognostic potential in cases of breast cancers and in other tumours.

Summing up, in the present study expression of CD24c-m has been confirmed to represent a strong unfavourable prognostic index in breast cancer. The remaining indices either have shown no relationship with the survival rates of the patients or their relation with survival was poor. On the other hand, examination of the entire panel of studied proteins has permitted to select groups of patients with the extremely good prognosis.

## Figures and Tables

**Table 1:** Patient and tumour characteristics

**Characteristics**	**No. (%)**	**Log-rank test**
All patients	104 (100)	
*Age (mean 56.2)*		—
⩽50	33 (32)	
>50–60	29 (28)	
>60	42 (40)	
		
*Menopause*		0.24
Premenopausal	30 (29)	
Postmenopausal	74 (71)	
		
*Grade*		0.21
2	71 (68)	
3	33 (32)	
		
*PT*		0.74
1	17 (16)	
2	86 (83)	
4	1 (1)	
		
*PN*		0.35
0	29 (28)	
1	75 (72)	
		
*PM*		—
0	104 (100)	
		
*Stage*		0.96
I	3 (3)	
IIa	40 (38)	
IIb	60 (58)	
IIIb	1 (1)	
		
*Histology*		—
Ductal	103 (99)	
Scirrhous	1 (1)	
		
*Therapy* a		—
Tamoxifen	70 (67)	
Radiotherapy	51 (49)	
Cyclophosphamide/methotrexate/5-fluorouracil	28 (27)	
Cyclophosphamide/adriamycin/5-fluorouracil	1 (1)	
Cyclophosphamide/adriamycin	1 (1)	
Progesterone	1 (1)	
Letrozol	1 (1)	

a Some patients received more than one special treatment.

**Table 2:** Relationships between studied proteins expressions and various clinicopathological factors

**Characteristics**	**ER *χ*^2^ test**	**pS2 *χ*^2^ test**	**MT *χ*^2^ test**	**CD24c-m *χ*^2^ test**	**CD24m *χ*^2^ test**
pT	0.0727	0.3948	0.2588	0.5050	0.6863
pN	0.3276	0.2717	0.0234	0.1499	0.7475
Stage	0.5195	0.2338	0.3691	0.2848	0.2620
Grade	0.9952	0.0587	0.1921	0.1972	0.4052
Age	0.7378^a^	0.3157^a^	0.1067^a^	0.7124	0.1698
Menopause status	0.1669	0.0553	0.6263	0.7628	0.1493
Progression	0.6606	0.0568	0.8573	**<0.0001**	0.7280
Death	0.9670	0.3604	0.9341	**<0.0001**	0.1558

ER, oestrogen receptor alpha; MT, metallothionein; CD24m, membranous CD24 expression; CD24c-m, cytoplasmic-membranous CD24 expression.

a Spearman's rank correlation.

Bold signifies *P*⩽0.05.

**Table 3:** Univariate analysis of relationships between expression of studied proteins and survival rates in the subgroups of the patients

**Patients groups**	**ER (IRS 0–2 *vs* 3–12) *P*-value**	**pS2 (IRS 0–2 *vs* 3–12) *P*-value**	**MT (IRS 0–4 *vs* 6–12) *P*-value**	**CD24c-m (negative *vs* positive) *P*-value**	**CD24m (negative *vs* positive) *P*-value**
*With tamoxifen, n=70*	*n*=35 *vs* *n*=35	*n*=38 *vs* *n*=32	*n*=24 *vs* *n*=46	*n*=43 *vs* *n*=27	*n*=41 *vs* *n*=29
OS	0.3107	0.2884	0.9408	**<0.0001** b	0.1668
PFS	0.9655	**0.0442** a	0.6148	**<0.0001** b	0.7100
					
*Without tamoxifen, n=34*	*n*=17 *vs* *n*=17	*n*=19 *vs* *n*=15	*n*=13 *vs* *n*=21	*n*=17 *vs* *n*=17	*n*=19 *vs* *n*=15
OS	0.0739^c^	0.7242	0.8852	0.0739^d^	0.6986
PFS	0.0739^c^	0.7242	0.8852	0.0739^d^	0.6725
					
*With radiotherapy, n=51*	*n*=30 *vs* *n*=21	*n*=27 *vs* *n*=24	*n*=15 *vs* *n*=36	*n*=28 *vs* *n*=23	*n*=30 *vs* *n*=21
OS	0.6239	0.3340	0.5025	**<0.0001** b	0.2349
PFS	0.6310	**0.0390** a	0.8549	**<0.0001** b	0.8650
					
*Without radiotherapy, n=53*	*n*=22 *vs* *n*=31	*n*=30 *vs* *n*=23	*n*=22 *vs* *n*=31	*n*=32 *vs* *n*=21	*n*=30 *vs* *n*=23
OS	0.4851	0.4622	0.5087	0.1252	0.4398
PFS	0.4732	0.4622	0.5087	0.1314	0.4284
					
*With chemotherapy, n=28*	*n*=15 *vs* *n*=13	*n*=17 *vs* *n*=11	*n*=7 *vs* *n*=21	*n*=13 *vs* *n*=15	*n*=17 *vs* *n*=11
OS	0.9171	0.6013	0.1320	**0.0054** b	0.4830
PFS	0.4950	0.4086	0.3182	**0.0023** b	0.8731
					
*Without chemotherapy, n=76*	*n*=37 *vs* *n*=39	*n*=40 *vs* *n*=36	*n*=30 *vs* *n*=46	*n*=47 *vs* *n*=29	*n*=43 *vs* *n*=33
OS	0.6846	0.3898	0.3600	**0.0005** b	0.2104
PFS	0.7277	0.0957	0.2505	**0.0007** b	0.6461

ER, oestrogen receptor alpha; MT, metallothionein; OS, overall survival; PFS, progression-free survival.

Bold signifies *P*⩽0.05.

a Higher expression correlates with better prognosis.

b Negative expression correlates with better prognosis.

c All patients with higher expression were alive or without progression.

d All patients without expression were alive or without progression.

**Table 4:** Multivariate analysis of relationships between expression of studied proteins and survival rates in the entire studied group and in subgroups of the patients

**Patient groups**	**ER *P*-value**	**pS2 *P*-value**	**MT *P*-value**	**CD24c-m *P*-value**	**CD24m *P*-value**
*Entire group, n=104*					
OS	0.349	0.574	0.330	**<0.001**	**0.029**
PFS	0.099	0.119	0.412	**<0.001**	0.149
					
*With tamoxifen, n=70*					
OS	0.759	0.155	0.319	**<0.001**	**0.011**
PFS	0.298	**0.007**	0.280	**<0.001**	**0.008**
					
*Without tamoxifen, n=34*					
OS	0.952	0.701	0.387	0.958	0.348
PFS	0.951	0.700	0.388	0.957	0.278
					
*With radiotherapy, n=51*					
OS	0.552	0.455	0.207	**<0.001**	0.056
PFS	0.160	**0.011**	0.156	**<0.001**	**0.020**
					
*Without radiotherapy, n=53*					
OS	0.652	0.646	0.735	0.249	0.563
PFS	0.631	0.649	0.733	0.263	0.545
					
*With chemotherapy, n=28*					
OS	0.233	0.690	0.064	0.954	0.638
PFS	0.072	0.664	0.097	0.946	0.901
					
*Without chemotherapy, n=76*					
OS	0.891	0.341	0.840	**0.001**	**0.037**
PFS	0.697	0.074	0.763	**0.001**	0.119

ER: oestrogen receptor alpha, MT: metallothionein, OS: overall survival, PFS: progression-free survival.

Bold signifies *P*⩽0.05.
